# Exploring the Active Constituents of *Andrographis paniculata* in Protecting the Skin Barrier and the Synergistic Effects with Collagen XVII

**DOI:** 10.3390/antiox14010118

**Published:** 2025-01-20

**Authors:** Heng Xu, Shiying Lan, Simin Lin, Anjing Wang, Yuanlin Luo, Jing Wang, Zhenzhong Yang

**Affiliations:** 1 Pharmaceutical Informatics Institute, College of Pharmaceutical Sciences, Zhejiang University, Hangzhou 310058, China; 22319145@zju.edu.cn (H.X.); 22419018@zju.edu.cn (S.L.); 22219038@zju.edu.cn (A.W.); luoyuanlin0928@zju.edu.cn (Y.L.); 2 Innovation Institute for Artificial Intelligence in Medicine, Zhejiang University, Hangzhou 310018, China; 3 Proya Cosmetics Co., Ltd., Hangzhou 310023, China; linsimin@proya.com (S.L.); wangjing48@proya.com (J.W.)

**Keywords:** skin homeostasis, Andrographis Herba, diterpene lactones, andrographolide, collagen XVII

## Abstract

*Andrographis paniculata* is mainly used to treat skin inflammations, wounds, and infections. In this study, Andrographis Herba, the aerial part of the plant, was proven to increase the viability of UVB-damaged HaCat cells and reduce reactive oxygen species levels. The chemical composition of Andrographis Herba extract (AHE) was analyzed using UPLC-Q-TOF-MS, and diterpene lactones were identified as its primary constituents. Then, the fraction of diterpene lactones was prepared and exhibited similar effects to AHE. AHE, its diterpene lactones component, and its representative constituent andrographolide all decreased the expression of IL-1β, IL-6, and CDKN1A. Furthermore, the protective effects of AHE and its active ingredients on UVB-damaged epidermal stem cells were investigated. Notably, the combined treatment with andrographolide and collagen XVII enhanced the viability of UVB-damaged epidermal stem cells, increased the expression of stemness markers integrin β1 and p63, and decreased the expression of the differentiation marker keratin 10. This combination demonstrated significant synergy in maintaining skin homeostasis, which provides evidences for the development of skin-protective products.

## 1. Introduction

Skin, as the largest organ of the human body, is the main defense barrier against harmful external environmental factors [1]. Photodamage from ultraviolet (UV, 10–400 nm) irradiation is common in daily life, especially in recent decades due to ozone layer depletion from atmospheric pollution [2]. Ultraviolet radiation of shorter wavelengths, such as ultraviolet C (UVC, 200–280 nm), is mostly filtered out by the atmosphere. Ultraviolet B (UVB, 280–320 nm) has a higher biological damage capacity compared to ultraviolet A (UVA, 320–400 nm), making it the primary source of UV-induced skin damage [3]. Skin homeostasis is a state where the skin is structurally and functionally balanced and stabilized, ensuring effective protection against external stimuli, moisture retention, temperature regulation, and damage repair [4]. The integrity of the skin barrier, along with a powerful capacity for self-renewal and repair, is essential for maintaining skin homeostasis.

Keratinocytes, located mainly in epidermis of the skin, account for about 90% or more of the epidermal cells on the surface of the skin as one of the most important components of the skin barrier [5]. Epidermal stem cells (ESCs), although accounting for only 1–5% of epidermal cells, are crucial for epidermal regeneration. Their strong capacity for self-renewal and differentiation allows them to continuously generate new keratinocytes to replace lost ones, thereby constantly renewing and repairing the skin barrier and maintaining skin homeostasis [6,7]. The combined effect of keratinocytes and ESCs plays an important role in the rapid repair of the skin barrier in the event of damage from external stimuli, thereby maintaining skin homeostasis. Studies have shown that UVB irradiation increases oxidative stress, inflammation, and cell death in skin cells, which in turn destroy the skin barrier and cause an imbalance in skin homeostasis [8,9,10].

Medicinal plants are a vast treasure trove and rich in bioactive compounds that play a crucial role in maintaining body homeostasis [11,12]. For instance, some plant extracts can effectively reduce inflammatory responses [13,14], protect cells from oxidative stress damage [15,16], and slow down the aging process of the skin [17,18]. They are also excellent resources for preventing UVB damage and maintaining skin homeostasis.

*Andrographis paniculata* is widely distributed in East and South Asia. Andrographis Herba (AH), the aerial part of *A. paniculata*, is mainly used as traditional medicine in the treatment of infection, pain, and other diseases [19,20,21,22], and the application of *A. paniculata* on the skin includes the treatment of skin infections, dermatitis, skin cancer, etc. [23,24,25]. It has been reported that the Andrographis Herba extract (AHE) protects keratinocytes by inhibiting oxidative stress and inflammation, suggesting the potential of AH for the treatment against epidermal damage [26]. However, whether AH can protect against UVB-induced damage to keratinocytes and ESCs has not been elucidated yet, and the related active constituents of AH need to be deciphered.

Collagen XVII (COL XVII) is a transmembrane protein. It plays an important role in repairing skin trauma by influencing stem cell migration, proliferation, and differentiation [27]. As an important component of ESCs hemidesmosomes, COL XVII maintains stem cell homeostasis and skin cell renewal to retain skin youthfulness [28]. COL XVII influences ESC competition, with higher expression levels anchoring cells to the basement membrane and promoting symmetrical division, thus preserving skin structure and integrity [29]. It is not clear whether COL XVII can play a protective role against UVB-induced ESCs damage.

Given the importance of maintaining skin homeostasis and protecting against damage, it is valuable to investigate how natural active ingredients from AHE are involved in skin health. To address this, AHE was prepared and isolated, and UVB damage models of the epidermal cells, including keratinocytes and ESCs, were used to identify the active ingredients in AHE that maintain skin homeostasis. Additionally, the synergistic effects of these representative constituents with COL XVII were studied.

## 2. Materials and Methods

### 2.1. Chemicals and Reagents

AH was collected from Zhejiang Province, China. Formic acid was obtained from Shanghai Aladdin Biochemical Technology Co., Ltd. (Shanghai, China). Methanol and acetonitrile were obtained from Merck KGaA (Darmstadt, Germany). Ultra-pure water was prepared with a Milli-Q system (Millipore, Bedford, MA, USA).

A cell counting kit-8 (CCK-8) was obtained from Biosharp (Hefei, China). A reactive oxygen species (ROS) assay kit was obtained from Uelandy (Suzhou, China). Dulbecco’s Modified Eagle’s Medium (DMEM) was obtained from WISENT INC (Saint-Jean-Baptiste, Canada). Keratinocyte serum-free medium (K-SFM) and 0.25% Trypsin-EDTA were obtained from Life Technologies Corporation (Grand Island, NY, USA). Dispase II was obtained from Sigma-Aldrich (St. Louis, MO, USA). Recombinant collagen type XVII was obtained from Jiangsu Chuangjian Medical Technology Co., Ltd. (Changzhou, China). Collagen type IV was obtained from Sigma-Aldrich (St. Louis, MO, USA). ChamQ SYBR qPCR Master Mix was obtained from Nanjing Vazyme Biotech Co., Ltd. (Nanjing, China). Easy RT MasterMix (DNase) was obtained from Zhejiang Easy-Do Biotech Co., Ltd. (Hangzhou, China). An RNA-Quick Purification Kit was obtained from ES Science. (Shanghai, China). A Protease Inhibitor Cocktail (EDTA-Free, catalog number HY-k0010) was obtained from MedChemExpress (Monmouth Junction, NJ, USA). Phenylmethylsulfonyl fluoride, a BeyoECL plus ultra-sensitive ECL Chemiluminescence Kit, a QuickBlock™ primary antibody dilution buffer, and a QuickBlock™ blocking buffer were obtained from Beyotime Biotechnology (Shanghai, China). Bovine serum albumin, Fraction V, heat shock isolation, and a 4% paraformaldehyde fix solution were obtained from BBI life sciences corporation (Shanghai, China). Immobilon^®^-P transfer membrane (0.45 µm pore size) was obtained from Merck Millipore (Tullagreen, Carrigtwohill, Co. Cork, Ireland). Integrin β1 antibody (catalog number 610468) was obtained from BD biosciences (San Jose, CA, USA). Keratin 19 polyclonal antibody (catalog number BS6018) was obtained from Bioworld Technology (Bloomington, MN, USA). Recombinant p63 antibody (catalog number ab124762) and recombinant keratin 10 antibody (catalog number ab76318) were obtained from Abcam (Cambridge, UK).

### 2.2. Sample Preparation

AH was cut into segments, dried at 60 °C for 2 h, and then extracted twice with a 50% ethanol aqueous solution (1:10, m/V) for 1 h. The extracts were combined and concentrated under reduced pressure. The AHE was then obtained through freeze-drying.

D101 macroporous resins were adopted to adsorb the AHE. After dynamic adsorption, elution was carried out with 5 bed volumes (BV) of water and 30% ethanol. Then, the macroporous resins were eluted with 5 BV of 70% ethanol, and the 70% ethanol eluents were collected. The eluents were concentrated under reduced pressure, and the Andrographis Herba diterpene lactones component (AHDL) was obtained after freeze-drying.

The AHE and AHDL were resuspended in 50% methanol at a concentration of 10 mg/mL. After centrifugation at 12,000 rpm for 10 min, supernatants were used for further analysis.

### 2.3. Sample Analysis

The analysis of AHE was performed on an ultra-performance liquid chromatography (UPLC) (Waters, Milford, MA, USA) equipped with a Triple TOF 6600+ mass spectrometer (AB SCIEX, Framingham, MA, USA). Chromatographic separation was carried out at 40 °C on an ACQUITY UPLC HSS T3 column (2.1 × 100 mm, 1.8 μm) with mobile phase A (0.1% formic acid-water) and mobile phase B (acetonitrile). The flow rate was 0.4 mL/min, and the injection volume was 10 μL. The elution conditions were optimized as follows: 0 min, 2% B; 20 min, 22% B; 26 min, 25% B; 31 min, 30% B; 35 min, 50% B; 40 min, 100% B; and 45 min, 100% B. The parameters for positive and negative modes were as follows: curtain gas of 35 psi; gas 1 (N_2_), 55 psi; gas 2 (N_2_), 55 psi; ion spray voltage, 5.5 kV (positive mode) and −4.5 kV (negative mode); collision energy, 10V; declustering potential, 80 V; ion source temperature, 550 °C (positive mode) and 550 °C (negative mode); and scan range, *m/z* 100–2000 Da.

The analysis of AHDL was performed on a high-performance liquid chromatography (HPLC) (Agilent Technologies, Santa Clara, CA, USA) at a wavelength of 203 nm. Other conditions were as described above.

### 2.4. Epidermal Stem Cells Culture and Characterization

ESCs were isolated and identified with the method described previously with slight modifications [30]. The procedure was approved by the Animal Ethics Committee of the Laboratory Animal Center, Zhejiang University (No. 30868). Neonatal SD rats were from the Zhejiang Academy of Medical Sciences (Hangzhou, China). Briefly, the back skin of a neonatal SD rat (typically 1–3 days old) was carefully removed in one piece. After incubating the skin tissue in 0.4% Dispase II solution at 4 °C overnight, the epidermis was separated from the dermis. The epidermal layer was digested using trypsin to obtain a single-cell suspension. Single cells were cultured in type IV collagen-coated culture flasks at 37 °C for 10 min, and the rapidly adhering cells were collected and cultured in K-SFM medium with 0.1 nM cholera toxin and 1% penicillin-streptomycin solution at 37 °C in 5% CO_2_.

The cells were fixed with paraformaldehyde in PBS for 10 min at room temperature, permeabilized with 0.1% Triton X-100 in PBS for 10 min to allow antibodies access to intracellular antigens, and blocked with a blocking solution for 20 min. The cells were identified to be ESCs with integrin β1, keratin 19, and keratin 10 [31] by immunofluorescence staining. Incubation with the primary antibody diluted in blocking solution was carried out overnight at 4 °C, followed by incubation with a fluorescently labeled secondary antibody diluted in blocking solution for 1 h at room temperature in the dark. Visualization and image capture were performed using a Leica TCS SP8 Confocal Microscope (Leica, Wetzlar, Germany).

### 2.5. Cell Viability Assay

HaCat cells were from the Cell Bank of the Shanghai Institute of Cell Biology, Chinese Academy of Sciences (Shanghai, China). HaCat cells were cultured in DMEM supplemented with 10% fetal bovine serum and 1% penicillin-streptomycin solution and maintained at 37 °C in a humidified atmosphere with 5% CO_2_.

The cells were seeded in 96-well plates at 1 × 10^4^ cells/well. When the cells in the 96-well plate reached 80% confluency, AHE, AHDL, and andrographolide (AG) were administered. The cells were irradiated with a UVB dose of 510 mJ/cm² and then incubated at 37 °C in a 5% CO_2_ incubator for 24 h. Cell viability was assessed using the CCK-8 assay.

ESCs were seeded in 96-well plates at 1 × 10^4^ cells/well. When the cells in the 96-well plate reached 80% confluency, AHE, AHDL, AG, and COL XVII were administered individually, and AG and COL XVII were also co-administered. The model was established with a UVB dose of 450 mJ/cm², followed by incubation at 37 °C in a 5% CO_2_ incubator for 24 h. Cell viability was assessed using the CCK-8 assay.

### 2.6. Measurement of Intracellular Production of ROS

HaCat cells were seeded in 96-well plates at a density of 1 × 10^4^ cells/well and pretreated with AHE, AHDL, and AG for 24 h. Nine hours after UVB irradiation, the fluorescent probe 2′,7′-dichlorodihydrofluorescein diacetate (DCFH-DA) was added for 30 min at 37 °C, and a fluorescence intensity of 2′,7′-dichlorofluorescein (DCF) was detected to reflect ROS levels. Images were taken using the ImageXpress^®^ Confocal HT.ai High-Content Imaging System (Molecular Devices, San Jose, CA, USA).

### 2.7. Reverse Transcription Quantitative Polymerase Chain Reaction (RT-qPCR)

Total RNA was isolated from the cells using an RNA-Quick Purification Kit and then converted into complementary DNA. The inflammatory and aging responses were assessed by measuring IL-1β, IL-6, and CDKN1A expression levels using an RT-qPCR (LightCycler 480 II, Roche Diagnostics GmbH) and analyzed with the 2^–ΔΔCt^ method. The primer sequences are presented in Table 1.

### 2.8. Western Blot

The cells were lysed using an IP buffer, and the proteins were separated using the sodium dodecyl sulfate-polyacrylamide gel electrophoresis procedure. The separated proteins were transferred from the gel to a polyvinylidene fluoride membrane using an electric current. The membrane was incubated with primary antibodies such as anti-integrin β1, anti-p63, and anti-keratin 10 that specifically bound to the target protein. After incubation, the membrane was washed to remove unbound primary antibodies, and then incubated with a secondary antibody. The membrane was washed again to eliminate any unbound secondary antibodies. After washing, the membrane was placed in a Bio-Rad chemiluminescence imaging system, an ECL detection reagent was added for development, and images were captured. The data were analyzed using ImageLab 5.2 software.

### 2.9. Statistical Analysis

All values are presented as mean ± SEM (n = 3). The significance of the differences between two groups was determined using a Student’s t-test. For comparisons involving more than three groups, a one-way ANOVA was employed. *p* values < 0.05 were considered statistically significant.

To evaluate the combination effect, the Bliss independent model was applied [32]. For each group, the effect, E, was calculated by Equation (1) [33].

(1) $$E=\frac{V_{t}-V_{m}}{V_{c}-V_{m}}$$

where *V_m_*, *V_t_*, and *V_c_* refer to the values of the UVB-damaged model group, the treatment group, and the control group, respectively.

The combined effects of AG and COL XVII were assessed using the combination index (CI), which was calculated by Equations (2) and (3). If CI is less than 1, it indicates that the combination effect exceeds the expected additive effect, demonstrating synergy [34].

(2) $$Expected\;additive\;effect=E_{A}+E_{B}-E_{A}E_{B}$$

(3) $$CI=\frac{E_{A}+E_{B}-E_{A}E_{B}}{E_{AB}}$$

where E_A_ and E_B_ represent the individual sample effects, while E_AB_ denotes the observed combined effect of AG and COL XVII.

## 3. Results

### 3.1. The Protective Effects of AHE on UVB-Damaged HaCat Cells

UVB radiation can destroy skin homeostasis, leading to sensitive skin, inflammation, photoaging, and other negative effects. Studies have shown that AH possesses anti-inflammatory and antioxidant properties [35,36,37]. HaCat cells are a spontaneously immortalized keratinocyte cell line derived from adult human skin, which exhibits excellent in vitro differentiation and proliferation capabilities. Herein, the protective effects of AH on UVB-damaged HaCat cells were studied.

**Figure 1:**
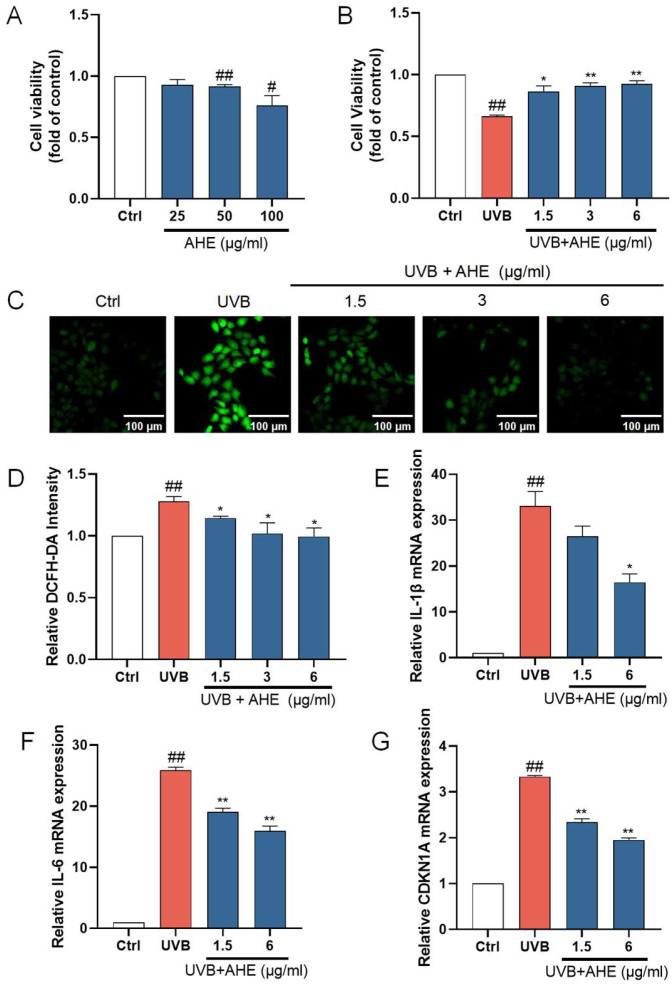
The protective effects of AHE on UVB-damaged HaCat cells. (**A**) The cells were incubated with AHE. Cell viability was measured by CCK-8 assay. (**B**) The cells were preincubated with AHE and irradiated with UVB (510 mJ/cm^2^). Cell viability was measured by CCK-8 assay. (**C**) The intracellular ROS in HaCat cells were assessed by DCFH-DA. The appearance of green fluorescence represents the intensity of the generated ROS. (**D**) The fluorescence intensity was quantified. The mRNA expression levels of (**E**) IL-1β, (**F**) IL-6, and (**G**) CDKN1A in HaCat cells were measured by RT-qPCR. ^#^ *p* < 0.05, ^##^ *p* < 0.01 vs. Ctrl group; * *p* < 0.05, ** *p* < 0.01 vs. UVB-damaged group.

HaCat cells were exposed to the AHE at a maximum concentration of 100 µg/mL, and cell viability was measured using the CCK-8 assay kit. The AHE at concentrations up to 25 µg/mL had no significant negative impact on the proliferation of HaCat cells (Figure 1A). To determine the appropriate UVB dosage, we examined how different UVB doses affect HaCat cell survival. As the UVB dose increased, the survival rate of HaCat cells continuously decreased. The IC_50_ for HaCat cells was determined to be 510 mJ/cm², and this dose was selected for subsequent experiments. While UVB irradiation significantly reduced the viability of HaCat cells, pretreatment with 1.5–6 µg/mL of AHE dose-dependently increased cell viability (Figure 1B).

UVB radiation induces the production of ROS within cells. Excessive ROS can lead to lipid peroxidation, protein degradation, DNA breakage, and the activation of pro-inflammatory pathways and oxidative stress, damaging cellular components and thereby accelerating skin barrier damage [38,39]. After UVB irradiation, the ROS levels in HaCat cells significantly increased. After treatment with 1.5–6 µg/mL AHE before UVB irradiation, the ROS levels in the cells significantly decreased (Figure 1C). This suggests that the AHE, within this concentration range, effectively reduces the accumulation of ROS induced by UVB, indicating notable antioxidant effects. The AHE appears to alleviate oxidative stress and cell damage caused by UVB through its antioxidant properties.

IL-1β and IL-6 are important inflammatory factors, and their increased expression can lead to an exacerbated inflammatory response in cells. The overexpression of inflammatory factors induces apoptosis in skin cells while inhibiting the proliferation of normal cells, leading to the decreased repair and regeneration capacity of skin tissue, thereby disrupting skin homeostasis [40]. To investigate the anti-inflammatory activity of the AHE on UVB-damaged HaCat cells, the mRNA levels of IL-1β and IL-6 in the cells were detected using an RT-qPCR. UVB irradiation significantly increased the expression of IL-1β and IL-6 in HaCat cells. Treatment with 1.5 and 6 µg/mL AHE prior to UVB irradiation resulted in a significant decrease in the expression of these factors and showed a dose-dependent effect (Figure 1E,F). This suggests that the AHE, within this concentration range, can effectively inhibit the inflammatory response induced by UVB.

CDKN1A is a cell cycle regulatory protein, and its increased expression can cause cell cycle arrest and cellular senescence. Cellular senescence is a state of permanent cell cycle arrest. High levels of CDKN1A impair tissue repair and regeneration, promoting aging and age-related diseases [41]. UVB irradiation significantly increased CDKN1A expression in HaCat cells. Treatment with 1.5 and 6 µg/mL AHE reduced CDKN1A expression in a dose-dependent manner (Figure 1G). This suggests that the AHE can effectively inhibit the cell cycle arrest and senescence induced by UVB.

### 3.2. Chemical Compositions Identification of AH

**Figure 2:**
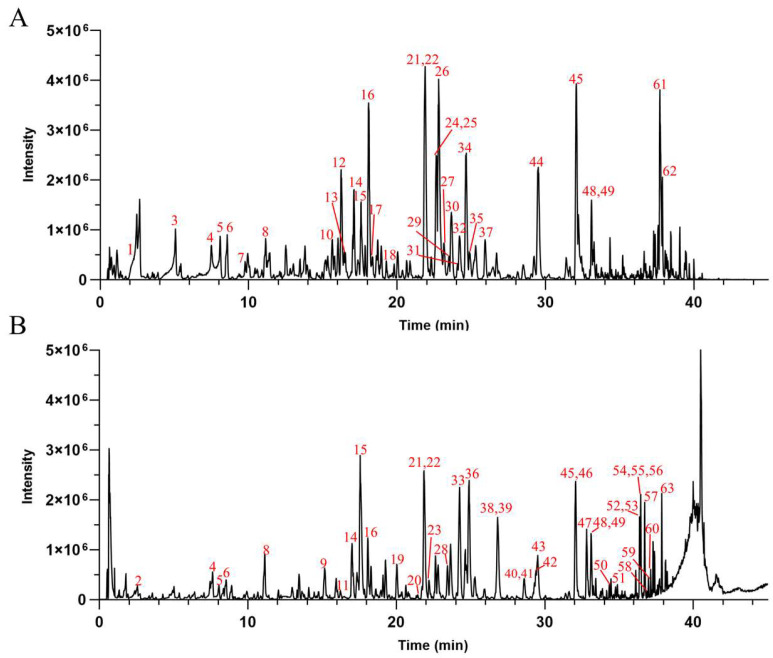
The base peak chromatograms of AHE obtained by UPLC-Q-TOF-MS in negative (**A**) and positive (**B**) ion modes.

UPLC-Q-TOF-MS was used for the chemical composition analysis of the AHE (Figure 2). Based on the results obtained from the UPLC-Q-TOF-MS analysis, combined with the data provided by the literature and databases, a total of 63 compounds were preliminarily identified in the AHE. Among the identified compounds, there were 28 diterpene lactones, 21 flavonoids, 4 polyphenols, 2 fatty acids, 1 phenylpropanoid, and 7 others, as detailed in Table 2. Diterpene lactones are the primary constituents of AH, making up a significant portion of its chemical composition. These compounds are known for their various pharmacological activities, including anti-inflammatory, antioxidant, and antimicrobial properties [42,43]. However, it has not been clarified whether diterpene lactones are the main active substances of AH responsible for protecting the skin barrier.

### 3.3. Isolation and Analysis of AHDL

**Figure 3:**
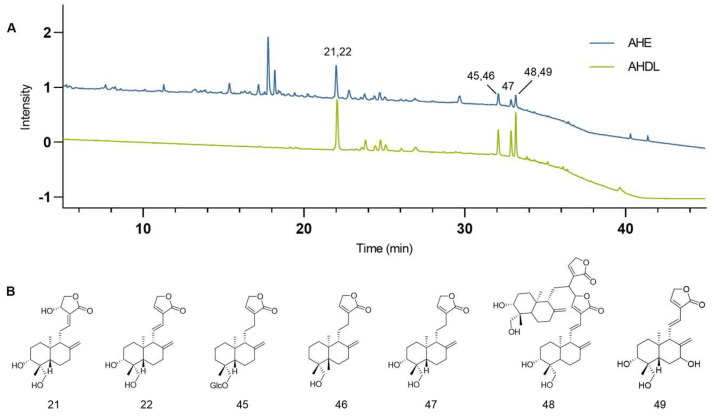
(**A**) The chromatograms of the AHE (blue) and AHDL (green) at 203 nm. (**B**) Representative diterpene lactone constituents in AHDL.

AHE was adsorbed using D101 macroporous resins, eluted with 70% ethanol, concentrated under reduced pressure, and freeze-dried to obtain AHDL. The AHDL was subjected to HPLC analysis and compared with the chromatogram of AHE at 203 nm. The main constituents of AHDL are diterpene lactones, such as AG, dehydroandrographolide, neoandrographolide, and so on (Figure 3).

### 3.4. The Protective Effects of AHDL on UVB-Damaged HaCat Cells

**Figure 4:**
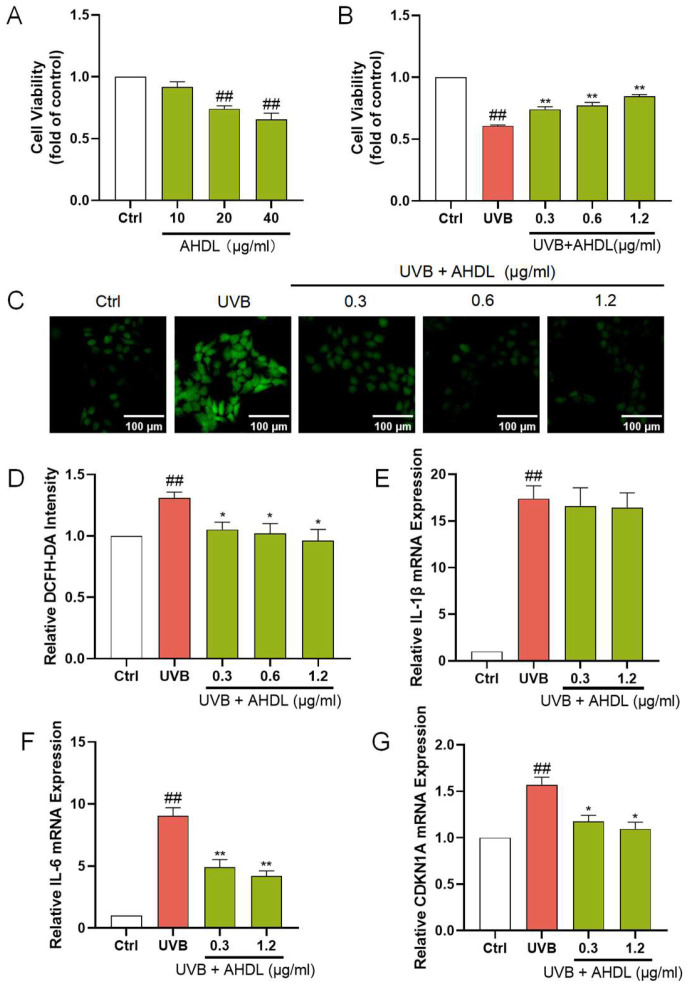
The protective effects of AHDL on UVB-damaged HaCat cells. (**A**) The cells were incubated with AHDL. (**B**) The cells were preincubated with AHDL and irradiated with UVB (510 mJ/cm^2^). (**C**) The intracellular ROS in HaCat cells was assessed by DCFH-DA. The appearance of green fluorescence represents the intensity of the generated ROS. (**D**) The fluorescence intensity was quantified. The mRNA expression levels of (**E**) IL-β, (**F**) IL-6, and (**G**) CDKN1A in HaCat cells were measured by RT-qPCR. ^##^ *p* < 0.01 vs. Ctrl group; * *p* < 0.05, ** *p* < 0.01 vs. UVB-damaged group.

AHDL is the primary component of AH. It remains unclear if AHDL is the main active substance in AH responsible for protecting the skin barrier. Hence, research is being conducted on the skin barrier protection effects of AHDL. AHDL at concentrations up to 10 µg/mL did not significantly impact the proliferation of HaCat cells (Figure 4A). The treatment of HaCat cells with 0.3, 0.6, and 1.2 µg/mL of AHDL before UVB irradiation led to a notable increase in cell viability (Figure 4B). Additionally, a significant reduction in ROS levels was observed (Figure 4C,D), indicating that AHDL can effectively mitigate the accumulation of ROS induced by UVB exposure. Furthermore, after treatment with 0.3 and 0.6 µg/mL, AHDL significantly decreased the expression of IL-6 and CDKN1A (Figure 4F,G) but had little effect on IL-1β (Figure 4E). AHDL exhibits effects comparable to the AHE, suggesting it may be the primary active component in AH responsible for protecting against UVB damage.

### 3.5. The Protective Effects of AG on UVB-Damaged HaCat Cells

**Figure 5:**
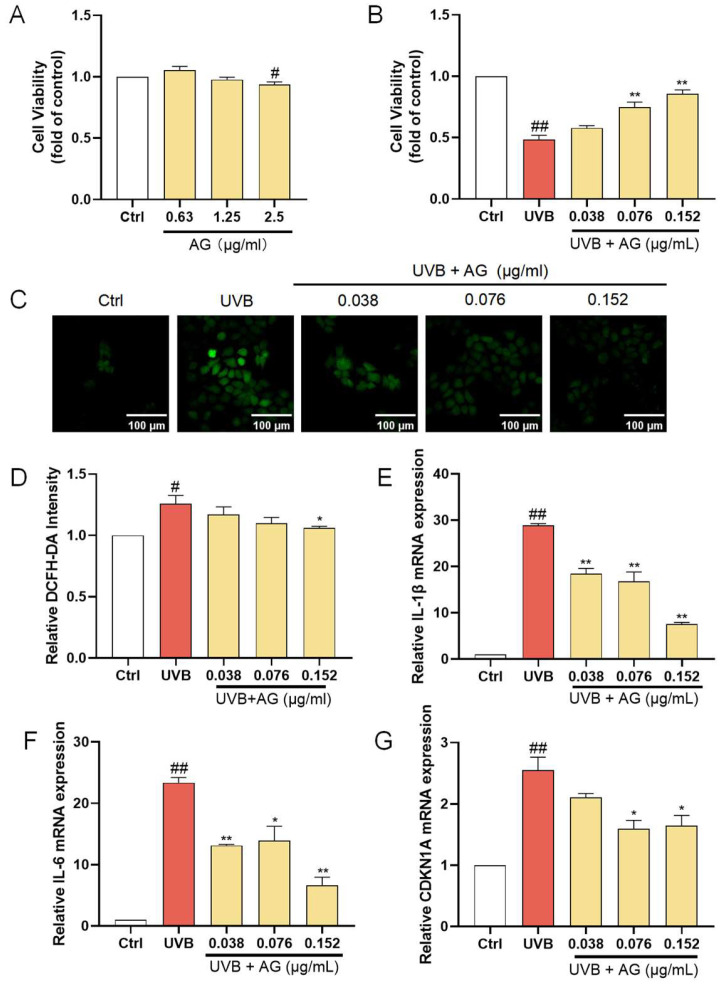
The protective effects of AG on UVB-damaged HaCat cells. (**A**) The cells were incubated with AG. (**B**) The cells were preincubated with AG and irradiated with UVB (510 mJ/cm^2^). (**C**) The intracellular ROS in HaCat cells was assessed by DCFH-DA. The appearance of green fluorescence represents the intensity of the generated ROS. (**D**) The fluorescence intensity was quantified. The mRNA expression levels of (**E**) IL-β, (**F**) IL-6, and (**G**) CDKN1A in HaCat cells were measured by RT-qPCR. ^#^ *p* < 0.05, ^##^ *p* < 0.01 vs. Ctrl group; * *p* < 0.05, ** *p* < 0.01 vs. UVB-damaged group.

AG, the most representative constituent in AHDL, should be the focus of subsequent activity research. At concentrations up to 1.25 µg/mL, AG did not significantly affect the proliferation of HaCat cells (Figure 5A). When HaCat cells were treated with 0.038, 0.076, and 0.152 µg/mL of AG before UVB irradiation, a remarkable increase in cell viability was observed (Figure 5B). Additionally, there was a significant reduction in ROS levels (Figure 5C,D), demonstrating the effectiveness of AG in mitigating UVB-induced ROS accumulation. Furthermore, AG significantly decreased the expression of IL-1β, IL-6, and CDKN1A (Figure 5E–G). These findings collectively indicate that AG may be a valuable compound for mitigating UVB-induced oxidative stress, inflammation, and aging in HaCat cells, potentially maintaining skin homeostasis and offering therapeutic benefits for skin health.

### 3.6. Identification of ESCs

**Figure 6:**
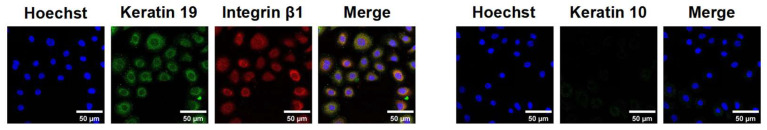
Identification of ESCs.

Integrin β1 is a transmembrane receptor crucial for cell adhesion, migration, and survival. Keratin 19 is a cytoskeletal protein in epithelial cells, particularly undifferentiated ESCs. Keratin 10 is a differentiation marker in differentiated keratinocytes. The high expression of integrin β1 and keratin 19, along with the near absence of keratin 10 (Figure 6), indicates that these cells exhibit the typical characteristics of ESCs, confirming that the isolated and extracted cells were primarily ESCs.

### 3.7. The Effects of AHE, AHDL, AG, and COL XVII on the Survival Rate of UVB-Damaged ESCs

**Figure 7:**
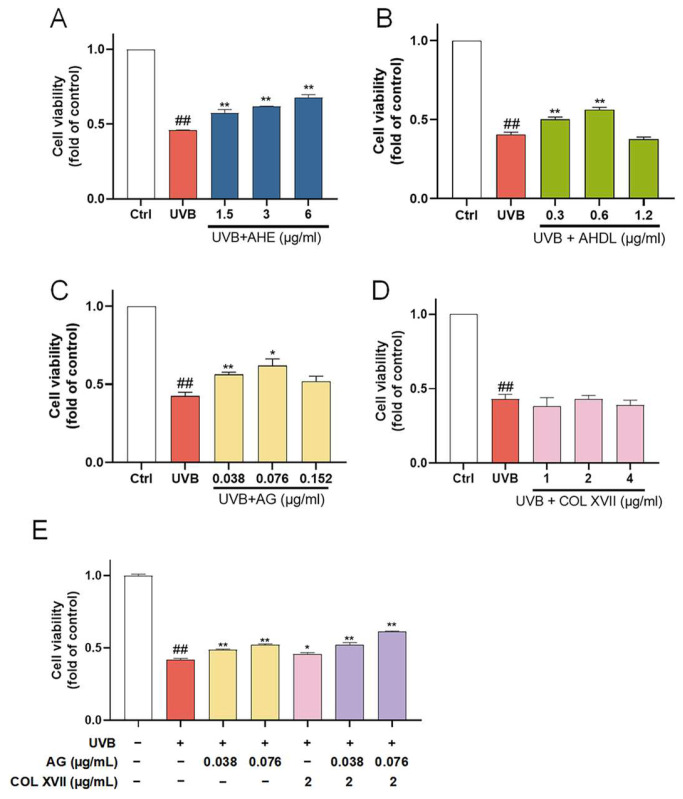
The protective effects of AHE, AHDL, AG, and COL XVII on UVB-damaged ESCs. The cells were preincubated with different treatments and irradiated with UVB (450 mJ/cm²). (**A**) AHE, (**B**) AHDL, (**C**) AG, (**D**) COL XVII, (**E**) Combination of AG and COL XVII. ^##^ *p* < 0.01 vs. Ctrl group; * *p* < 0.05, ** *p* < 0.01 vs. UVB-damaged group.

The ESC damage model was established by UVB irradiation. When the dose is 450 mJ/cm², the cell survival rate is about 50%. UVB irradiation significantly reduced the viability of ESCs, which was reversed when pretreated with AHE, AHDL, or AG (Figure 7A–C). COL XVII is a transmembrane protein essential for skin aging and wound healing. It maintains skin integrity by binding the epidermis and dermis, ensuring elasticity, repair, and protection against damage. But COL XVII did not have a significant protective effect on the cell viability of UVB-damaged ESCs at the tested concentrations (Figure 7D).

When co-treated with AG and COL XVII, the cell survival rate increased compared to AG and COL XVII alone (Figure 7E). This suggests that AG and COL XVII have a synergistic effect, which can more effectively improve the survival rate of UVB-damaged cells.

### 3.8. The Synergistic Effects of AG and COL XVII on UVB-Damaged ESCs

In order to further evaluate the synergistic protective effect of AG and COL XVII on UVB-induced ESC damage, we used western blotting to assess the expression levels of the stemness proteins including integrin β1 and p63.

**Figure 8:**
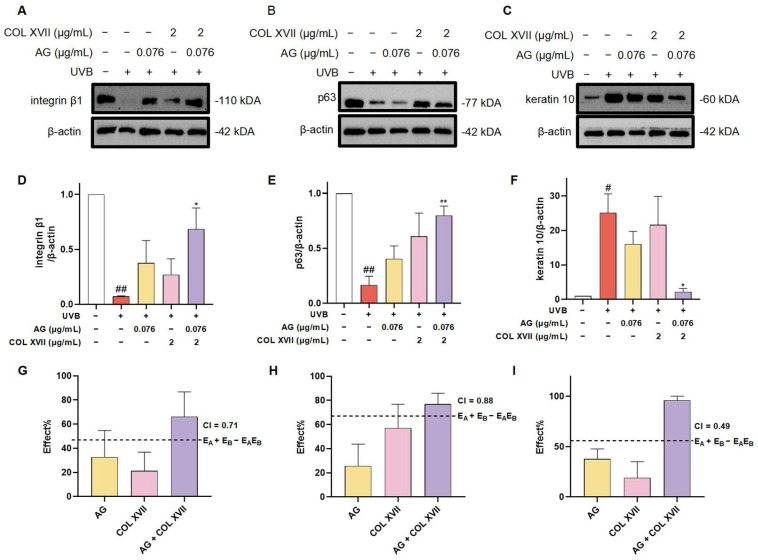
Changes in the expression of ESC markers of UVB-damaged ESCs after combined treatment with AG and COL XVII and the exploration of the combination index (CI). The protein expression levels of integrin β1 (**A**,**D**), p63 (**B**,**E**), and keratin 10 (**C**,**F**) were measured by western blotting. Comparison of the observed effects and additive effects of AG and COL XVII on integrin β1 (**G**), p63 (**H**), and keratin 10 (**I**). ^#^ *p* < 0.05, ^##^ *p* < 0.01 vs. Ctrl group; * *p* < 0.05, ** *p* < 0.01 vs. UVB-damaged group.

Integrin β1, widely expressed at high levels in the basement membranes of ESCs, regulates epidermal stem cell proliferation and differentiation by mediating the adhesion between cells and the basement membrane maintaining skin homeostasis [44]. The immunoblotting results showed that expression of integrin β1 in UVB-irradiated ESCs was significantly decreased, whereas there was a restoration of integrin β1 expression with the treatment of AG or COL XVII, though there was no significant difference. When co-treated with AG and COL XVII, the expression of integrin β1 was greatly restored, suggesting that AG and COL XVII combined administration can protect UVB-induced ESC damage to maintain skin homeostasis by increasing integrin β1 expression (Figure 8A,D,G).

It is well known that the powerful self-renewal capacity of ESCs is essential for the maintenance of skin homeostasis. p63, a specific marker for ESCs in the basement membrane of the skin, is responsible for the regulation of cell proliferation and differentiation, the inhibition of apoptosis, and the maintenance of stem cell self-renewal capacity [45]. It has been shown that p63-depleted ESCs lose their proliferative capacity and move towards terminal differentiation [46]. Noticeably, the expression of p63 was significantly decreased in UVB-damaged ESCs, which was distinctly upregulated with AG and COL XVII combined treatment compared with the individual use of AG or COL XVII (Figure 8B,E,H). Taken together, these results suggest that the cotreatment of AG and COL XVII can largely reverse UVB-induced decreases in p63 protein expression in ESCs to retain the self-renewal capacity of ESCs helping keep skin homeostasis.

The balance of epidermal cell proliferation and differentiation plays a crucial role in maintaining skin homeostasis, while UVB irradiation severely disturbs the balance of epidermal cell proliferation and differentiation [47]. To further investigate the effect of AG and COL XVII combination on UVB-induced proliferation and the differentiation imbalance of ESCs, we assessed the expression level of the epidermal differentiation marker, keratin 10. There was an extremely salient increase in the expression of keratin 10 in UVB-damaged ESCs. However, it was very noteworthy that the synergy of AG and COL XVII reduced the expression of keratin 10 compared to single effects (Figure 8C,F,I).

These findings indicate that AG and COL XVII combination administration could reverse UVB-induced imbalance in ESC proliferation and differentiation. The effects of the combined usage of AG and COL XVII were compared side-by-side with the effects of single administration. As anticipated, when used together, AG and COL XVII showed further increased effects in maintaining skin homeostasis than that of each single treatment, demonstrating a CI value of less than 1 and indicating significant synergy beyond simply additive effects.

## 4. Discussion

The skin is the first line of body defense, playing a crucial role in protecting against external pathogens and maintaining cellular homeostasis [48,49]. Homeostatic imbalance is a common symptom in various skin diseases. Exposure to high levels of UV radiation could cause oxidative stress and inflammation. This disruption in skin homeostasis results in a range of problems, including aging, sensitivity, and dryness. Therefore, maintaining skin homeostasis is essential to skin health. Studies showed that *A. paniculata* extract protected dermal fibroblasts and keratinocytes from inflammation and oxidative stress [26,50]. In this study, we explored the active ingredients in *A. paniculata* and further investigated the effects of its representative active constituent and COL XVII, a protein that plays a crucial role in maintaining skin integrity, on skin homeostasis.

UVB exposure increases ROS levels in HaCat cells, which in turn causes cell inflammation, senescence, and death [51,52,53]. We investigated the effects of AHE on UVB-irradiated HaCat cells and found that it dose-dependently improved cell survival, reduced ROS content, and decreased the expression of IL-1β, IL-6, and CDKN1A, indicating the protective effect of the AHE. The UPLC-Q-TOF-MS analysis of AHE identified 63 compounds, with 28 being diterpene lactones. Then, AHDL was prepared and indicated to be the main active substance of the AHE in protecting against UVB damage. The HPLC analysis revealed AG as the main constituent of AHDL. The anti-photodamage effect demonstrated by AG on keratinocytes is consistent with its reported role in reducing UV-induced acute photodamage in the skin [54]. Therefore, AG is the representative active constituent of AHDL. UVB activates the mitogen-activated protein kinase (MAPK) and nuclear factor kappa-B (NF-κB) pathways by the accumulation of ROS, which increases the expression of various inflammatory cytokines and matrix metalloproteinases (MMPs) [55,56]. The overexpression of MMPs then induces collagen degradation. Studies have shown that AG exerts anti-inflammatory effects on lipopolysaccharide-induced RAW264.7 cells by inhibiting the activation of the MAPK and NF-κB signaling pathways [57]. AG may inhibit UVB-induced damage by inhibiting the MAPK and NF-κB pathways. ROS induced by UVB can increase the expression of CDKN1A through stimulating the p38/p53 signaling pathway, leading to cell cycle arrest [58]. AG may reduce cell cycle arrest and maintain the balance of skin homeostasis by decreasing the expression of CDKN1A.

ESCs play an important role in maintaining skin homeostasis. If ESCs are exposed to external stimuli, i.e., repetitive UVB radiation, the structure and function of the epidermal barrier might be damaged, leading to an imbalance in skin homeostasis. The development of active substances that protect ESCs from UVB damage is of great importance. UVB-induced damage to ESCs causes cell death, decreased stemness, and abnormal proliferation and differentiation [59,60]. The AHE could maintain the stemness of epidermal stem cells by upregulating integrin β1 and VEGF expressions [61]. Based on previous results, we hypothesize that AG is likely to have activity in restoring the stemness of ESCs. Integrin β1, widely expressed at high levels in basement membrane ESCs, regulates ESC proliferation and differentiation by mediating adhesion between cells and the basement membrane to sustain skin homeostasis. p63, a specific marker for ESCs in the basement membrane of the skin, is responsible for the inhibition of apoptosis and the maintenance of stem cell self-renewal capacity. UVB irradiation severely disturbs the balance of epidermal cell proliferation and differentiation [47]. Therefore, we evaluated the activity of AG with COL XVII in a UVB-damage model of ESCs. Our results showed that AG rescued the UVB-induced decrease in cell viability and possessed similar activity to the AHE and AHDL. Notably, the combination of AG and COL XVII more effectively counteracts the UVB-induced decrease in ESCs stemness markers and increase in differentiation protein compared to each individual treatment, demonstrating significant synergy. AG may protect ESCs from UVB-induced damage through antioxidant and anti-inflammatory pathways. COL XVII may promote ESC competition by reducing UVB-induced hemidesmosomes degradation to reverse UVB-induced ESC homeostasis imbalance [29]. Further research is still needed to validate these hypotheses. As anticipated, AG and COL XVII, when used together, showed significantly greater effects in maintaining skin homeostasis than each treatment alone. Therefore, the combination of AG and COL XVII appears to be a promising composition for protecting the skin barrier. Further in vivo studies are needed as well as in-depth explorations of the mechanism of action, which will support the development of skincare products based on this combination.

## 5. Conclusions

In UVB-damaged cell models, we systematically evaluated the protective effects of AH at three levels: the whole extract, the component, and the active constituent. AHDL and AG in AH are key active ingredients that potentially protect the skin barrier through anti-inflammatory and antioxidative stress pathways. The combination of AG with COL XVII synergistically enhances skin barrier function and maintains skin homeostasis, offering evidences for the development of products aimed at skin barrier protection.

## Figures and Tables

**Table 1:** Primer sequences for RT-qPCR assays and genes accession numbers.

Name	Forward Primer Sequence (5′–3′)	Reverse Primer Sequence (5′–3′)
IL-1β (NM_000576.3)	CCACAGACCTTCCAGGAGAATG	GTGCAGTTCAGTGATCGTACAGG
IL-6 (XM_054358146.1)	TGACAAACAAATTCGGTACATCCTC	GTGCCTCTTTGCTGCTTTCAC
CDKN1A (NM_001374511.1)	TGTCCGTCAGAACCCATGC	AAAGTCGAAGTTCCATCGCTC
GAPDH (NM_001357943.2)	AATGAAGGGGTCATTGATGG	AAGGTGAAGGTCGGAGTCAA

**Table 2:** Chemical compositions analysis of Andrographis Herba.

No.	t_R_ (Min)	Identification	Formula	Detected *m/z*	Error (ppm)	Main MS/MS Fragments
1	1.96	Guanosine	C_10_H_13_N_5_O_5_	282.0847 [M − H]^−^	1.1	150.0346, 133.0085
2	2.54	Ethylparaben	C_9_H_10_O_3_	167.0703 [M + H]^+^	0.2	121.0640, 81.0334
3	5.06	Neochlorogenic acid	C_16_H_18_O_9_	353.0887 [M − H]^−^	2.5	191.0474, 179.0264, 135.0389
4	7.50	Chlorogenic acid	C_16_H_18_O_9_	353.0887 [M − H]^−^	2.5	191.0477, 135.0382
5	8.07	1-O-Caffeoylquinic acid	C_16_H_18_O_9_	353.0887 [M − H]^−^	2.5	191.0476, 173.0377, 135.0390
6	8.54	6-epi-8-O-Acetylharpagide	C_17_H_26_O_11_	451.1466 [M + FA − H]^−^	2.0	139.0331, 121.0232
7	9.77	3-p-Coumaroyl quinic acid	C_16_H_18_O_8_	337.0934 [M − H]^−^	1.5	191.0471, 173.0367, 119.0443
8	11.14	6,8-di-C-β-D-glucosylchrysin	C_27_H_30_O_15_	593.1524 [M − H]^−^	2.0	383.0592, 353.0495
9	15.17	Scutellarin /Luteolin-7-glucuronide	C_21_H_18_O_12_	463.0861 [M + H]^+^	−2.2	287.0561
10	15.63	14-Deoxy-11-hydroandrographolide or isomer	C_19_H_28_O_5_	381.1928 [M + FA − H]^−^	2.4	337.1870, 293.1190
11	16.22	Andrographidine B	C_23_H_24_O_12_	493.1324 [M + H]^+^	−3.4	331.0818
12	16.25	14-Deoxy-11-hydroandrographolide or isomer	C_19_H_28_O_5_	381.1927 [M + FA − H]^−^	2.2	337.1867
13	16.43	3,4-di-O-Caffeoylquinic acid	C_25_H_24_O_12_	515.1201 [M − H]^−^	1.2	353.0717, 191.0469, 179.0267, 135.0386
14	17.09	Andrographiside	C_26_H_40_O_10_	557.2619 [M + FA − H]^−^	2.8	493.2223, 331.1771, 161.0379
15	17.59	Apigenin-7-O-β-D-glucuronide	C_21_H_18_O_11_	445.0785 [M − H]^−^	1.9	269.0333, 117.0286
16	18.09	12S-Hydroxyandrographolide	C_20_H_32_O_6_	367.2136 [M − H]^−^	2.7	349.1861, 331.1761, 307.1776
17	18.34	3,4-di-O-Caffeoylquinic acid isomer	C_25_H_24_O_12_	515.1201 [M − H]^−^	1.2	353.0711, 191.0469, 173.0375
18	19.28	5,2′,6′-Trihydroxy-7-methoxyflavone 2′-O-β-D-glucoside	C_22_H_22_O_11_	461.1096 [M − H]^−^	1.4	299.0433, 284.0203
19	20.03	Scutellarin /Luteolin-7-glucuronide	C_21_H_18_O_12_	463.0860 [M + H]^+^	−2.4	287.0559
20	21.35	Andrographidine F	C_25_H_28_O_13_	537.1580 [M + H]^+^	−4.2	375.1080, 197.0443
21	21.88	Andrographolide	C_20_H_30_O_5_	395.2086 [M + FA − H]^−^	2.7	331.1764, 287.1889
22	21.88	Dehydroandrographolide	C_20_H_28_O_4_	331.1922 [M − H]^−^	2.2	303.1814, 301.1649
23	22.21	Andrographidine E or isomer	C_24_H_26_O_11_	491.1533 [M + H]^+^	−3.0	329.1026, 299.0553
24	22.56	12,13-Dihydroandrographolide	C_20_H_32_O_5_	351.2187 [M − H]^−^	2.9	333.1924, 321.1920, 157.0950
25	22.65	3,13,14,19-Tetrahydroxy-ent-labda-8(17),11-dien-16,15-olide	C_20_H_30_O_6_	365.1981 [M − H]^−^	3.1	321.1930
26	22.81	Andrographic acid	C_20_H_28_O_6_	363.1827 [M − H]^−^	3.8	319.1775
27	23.18	3-O-β-D-Glucopyranosylandrographolide	C_26_H_40_O_10_	511.2561 [M − H]^−^	2.4	467.2438, 305.1983
28	23.44	4′,5-Dihydroxy-7,8-dimethoxyflavone	C_17_H_14_O_6_	315.0860 [M + H]^+^	−1.0	299.0546, 282.0520, 271.0594
29	23.44	7,8-Dimethoxy-2′-hydroxy-5-O-β-D-glucopyranosyloxyflavone	C_23_H_24_O_11_	475.1255 [M − H]^−^	1.9	313.0565, 283.0107
30	23.66	Isoandrographolide	C_20_H_30_O_5_	395.2087 [M + FA − H]^−^	3.0	331.1758, 287.1885, 239.1690
31	24.08	3α,19-Dihydroxy-14,15,16-trinor-ent-labd-8(17),11-diene-13-oic acid	C_17_H_26_O_4_	293.1772 [M − H]^−^	4.7	157.0947
32	24.26	Andrographidine A or isomer	C_23_H_26_O_10_	461.1463 [M − H]^−^	2.1	299.0779, 269.0324, 241.0382
33	24.25	5-Hydroxy-7,8-Dimethoxyflavanone or isomer	C_17_H_16_O_5_	301.1069 [M + H]^+^	−0.5	197.0450, 182.0213, 136.0158
34	24.64	Andropanoside	C_26_H_40_O_9_	541.2670 [M + FA − H]^−^	2.9	495.2381, 333.1918, 161.0379
35	24.90	Andrographidine A or isomer	C_23_H_26_O_10_	461.1465 [M − H]^−^	2.6	299.0785, 269.0328, 241.0383
36	24.90	5-Hydroxy-7,8-Dimethoxyflavanone or isomer	C_17_H_16_O_5_	301.1068 [M + H]^+^	−0.8	197.0450, 182.0214, 136.0157
37	25.94	3-O-β-D-Glucosyl-14-deoxy-11,12- didehydroandrographiside	C_26_H_38_O_9_	539.2517 [M + FA − H]^−^	3.5	493.2222, 331.1768
38	26.83	Andrographidine C	C_23_H_24_O_10_	461.1430 [M + H]^+^	−2.7	299.0925, 284.0686, 255.0660
39	26.83	Apigenin 7,4′ -dimethyl ether	C_17_H_14_O_5_	299.0911 [M + H]^+^	−1.0	283.0601, 255.0655, 238.0624
40	28.61	Andrographidine E or isomer	C_24_H_26_O_11_	491.1538 [M + H]^+^	−2.0	329.1028, 299.0550
41	28.61	5-Hydroxy-7,2′,5′-Trimethoxyflavone or isomer	C_18_H_16_O_6_	329.1017 [M + H]^+^	−0.8	313.0706, 299.0544
42	29.41	Quercetin tetramethyl(3’,4’,5,7) ether	C_19_H_18_O_7_	359.1115 [M + H]^+^	−2.9	329.0656
43	29.41	7,8,2′,5′-Tetramethoxy-5-β-Dglucopyranosyloxyflavone or Andrographidine D	C_25_H_28_O_12_	521.1636 [M + H]^+^	−3.4	359.1132, 329.0664
44	29.51	14-Deoxy-11-oxoandrographolide	C_20_H_28_O_5_	347.1881 [M − H]^−^	4.9	303.1833, 301.1679, 255.1641
45	32.08	Neoandrographolide	C_26_H_40_O_8_	525.2720 [M + FA − H]^−^	2.8	317.1945, 101.0194
46	32.08	Andrograpanin	C_20_H_30_O_3_	319.2264 [M + H]^+^	−1.2	301.2166, 289.2164, 205.1223
47	32.82	14-Deoxyandrographolide	C_20_H_30_O_4_	669.4343 [2M + H]^+^	−2.7	299.2016, 287.2018, 259.1700
48	33.13	Bisandrographolide B	C_40_H_56_O_8_	665.4031 [M + H]^+^	−2.5	315.1957, 297.1854, 285.1855, 257.1542
49	33.10	7-Hydroxy dehydroandrographolide	C_20_H_28_O_5_	347.1882 [M − H]^−^	5.2	303.1833, 255.1640
50	34.34	Bisandrographolide C	C_40_H_56_O_8_	665.4024 [M + H]^+^	−3.6	647.3955, 629.3845, 205.1582
51	34.88	Panicolin	C_17_H_14_O_6_	315.0858 [M + H]^+^	−1.6	299.0546, 282.0516, 271.0601
52	36.34	Bisandrographolide A	C_40_H_56_O_8_	665.4028 [M + H]^+^	−3.0	629.3805, 617.3828, 599.3733
53	36.38	7-O-Methyldihydrowogonin isomer	C_17_H_16_O_5_	301.1066 [M + H]^+^	−1.5	197.0450, 182.0214, 164.0108
54	36.41	Bisandrographolide F	C_40_H_56_O_8_	665.4024 [M + H]^+^	−3.6	629.3837, 611.3737, 599.3726
55	36.46	7-O-Methyldihydrowogonin	C_17_H_16_O_5_	301.1068 [M + H]^+^	−0.8	197.0451, 182.0215, 164.0106
56	36.54	5-Hydroxy-3,7,8,2′-tetramethoxyflavone	C_19_H_18_O_7_	359.1113 [M + H]^+^	−3.4	329.0653, 183.0277
57	36.72	5-Hydroxy-7,8-dimethoxyflavone	C_17_H_14_O_5_	299.0909 [M + H]^+^	−1.7	283.0605, 267.0657,255.0660
58	36.88	5-Hydroxy-7,8,2′,5′-tetramethoxyflavone	C_19_H_18_O_7_	359.1114 [M + H]^+^	−3.1	329.0649, 311.0541
59	36.98	Andrographin	C_18_H_16_O_6_	329.1014 [M + H]^+^	−1.7	299.0545, 285.0750
60	37.14	5-Hydroxy-7,8,2′,3′-tetramethoxyflavone	C_19_H_18_O_7_	359.1119 [M + H]^+^	−1.8	329.0667, 286.0477
61	37.71	Chaetoglobosin K/Chaetoglobosin L	C_34_H_40_N_2_O_5_	555.2873 [M − H]^−^	1.5	224.9982, 164.9794, 80.9621
62	37.87	(10E,12E)-9-Hydroxyoctadeca-10,12-dienoic acid	C_18_H_32_O_3_	295.2299 [M − H]^−^	6.9	277.2057, 195.1305, 171.0953
63	37.87	Linolenic acid	C_18_H_30_O_2_	279.2318 [M + H]^+^	−0.2	95.0858, 81.0702, 67.0548

## Data Availability

All the data used to support the findings of this study are available from the corresponding author upon reasonable request.
